# Effect of TheraCyte-encapsulated parathyroid cells on lumbar fusion in a rat model

**DOI:** 10.1007/s00586-012-2418-5

**Published:** 2012-07-06

**Authors:** Sung-Hsiung Chen, Shun-Chen Huang, Chun-Chung Lui, Tzu-Ping Lin, Fong-Fu Chou, Jih-Yang Ko

**Affiliations:** 1 Department of Orthopaedic Surgery, Kaohsiung Chang Gung Memorial Hospital, Chang Gung University College of Medicine, Kaohsiung, Taiwan; 2 Department of Anatomic Pathology, Kaohsiung Chang Gung Memorial Hospital, Chang Gung University College of Medicine, Kaohsiung, Taiwan; 3 Department of Diagnostic Radiology, Kaohsiung Chang Gung Memorial Hospital, Chang Gung University College of Medicine, Kaohsiung, Taiwan; 4 Department of General Surgery, Kaohsiung Chang Gung Memorial Hospital, Chang Gung University College of Medicine, #123 Ta-Pei Rd., Niao-Sung Dist., Kaohsiung, Taiwan

**Keywords:** Animal models, TheraCyte parathyroid cells, Lumbar fusion

## Abstract

**Introduction:**

Implantation of TheraCyte 4 × 10^6^ live parathyroid cells can increase the bone marrow density of the spine of ovariectomized rats. There has been no published study examining the effect of such implantation on spinal fusion outcomes. The purpose of this study was to examine the effect of TheraCyte-encapsulated parathyroid cells on posterolateral lumbar fusions in a rat model.

**Materials and methods:**

Forty Sprague-Dawley rats underwent single-level, intertransverse process spinal fusions using iliac crest autograft. The rats were randomly assigned to two groups: Group 1 rats received sham operations on their necks (control; *N* = 20); Group 2 rats were implanted with TheraCyte-encapsulated 4 × 10^6^ live parathyroid cells into the subcutis of their necks (TheraCyte; *N* = 20). Six weeks after surgery the rats were killed. Fusion was assessed by inspection, manual palpation, radiography, and histology. Blood was drawn to measure the serum levels of calcium, phosphorus, and intact parathyroid hormone (iPTH).

**Results:**

Based on manual palpation, the control group had a fusion rate of 33 % (6/18) and the TheraCyte group had a fusion rate of 72 % (13/18) (*P* = 0.044). Histology confirmed the manual palpation results. Serum iPTH levels were significantly higher in the TheraCyte group compared with the control group (*P* < 0.05); neither serum calcium nor phosphorus levels were significantly different between the two groups.

**Discussion:**

This pilot animal study revealed that there were more fusions in rats that received TheraCyte-encapsulated 4 × 10^6^ live parathyroid cells than in control rats without significant change in serum calcium or phosphorus concentrations. As with any animal study, the results may not extrapolate to a higher species. Further studies are needed to determine if these effects are clinically significant.

## Introduction

It has long been believed that good therapeutic outcomes with regard to degenerative disease of the lumbar spine are achieved by fusion after posterior fusion [1]. Failure to achieve solid fusion may lead to loss of alignment, instability, pain, and potential neurologic injury. Augmentation with autologous bone graft is the current gold standard procedure to achieve lumbar solid fusion [2]. However, use of autograft is limited by supply and there is significant donor site morbidity [3]. Many attempts have been undertaken to minimize the need to harvest autograft and maximize the effect of autograft or bone graft alternatives. A potential alternative for facilitating fusion is the administration of systemic medication that has known anabolic effects on bone. Parathyroid hormone (PTH) is one such candidate protein. Daily injection appears to induce a net increase in bone formation [2–6], assist fracture healing [7–9], and facilitate bone formation and fusion rates in the rat posterolateral lumbar fusion model [10, 11]. However, daily injection of PTH is inconvenient and troublesome. Another option is transplantation of parathyroid tissues, but the main obstacle with this option is immunological rejection [12, 13]. We as well as others have found that using a microencapsulate of human parathyroid cells significantly increase PTH secretion in vitro and in rats without pharmacological immunosuppression [14–16]. Following the implantation of TheraCyte-encapsulated parathyroid cells in rats, we found that the parathyroid cells could survive and secrete intact PTH (iPTH) for up to 3 months [14]. No lymphocyte infiltration was found within the TheraCyte device. Implantation of TheraCyte 4 × 10^6^ live parathyroid cells can function very well and increase bone marrow density (BMD) in the lumbar spine for up to 3 months or longer. There has been no published study examining its effect on spinal fusion outcomes. The purpose of this study is to examine the effect of TheraCyte-encapsulated parathyroid cells on posterolateral lumbar fusion in a rat model.

## Materials and methods

Forty Sprague-Dawley rats approximately 10 weeks of age and 220–280 g in weight were selected for this study. Anesthesia was induced with ketamine and xylocaine intraperitoneally, and perioperative antibiotics were administered subcutaneously. The rats were placed in the prone position and prepared in standard surgical fashion. L4–L5 posterolateral fusions were performed [17, 18]. The spine was approached (Wiltse approach) through a single midline skin incision and two paramedian fascial incisions. The spinal level was determined with reference to the iliac crests. After exposure, the transverse process was decorticated, bone graft was harvested from both iliac crests, and fascial incisions were made over the iliac crests. A rongeur was used to harvest approximately 0.1–0.2 cc of morselized corticocancellous bone from both iliac crests. The wounds were irrigated, and the harvested graft was placed into the fusion levels. We put the grafts under the fascia of the paraspinal skeletal muscle after the fascia was opened. Then the fascia was sutured. So the graft would remain at the fusion site. At the same time, the rats were randomly assigned to one of two groups: Group 1 rats underwent sham operations on their necks (control group; *n* = 20); Group 2 rats were implanted with TheraCyte-encapsulated 4 × 10^6^ live parathyroid cells into the subcutis of their necks (TheraCyte group; *n* = 20). The Animal Use and Care Committee of our hospital approved these procedures.

### Preparation of PTH cells [14]

Parathyroid glands were obtained from patients who had undergone surgery for symptomatic secondary hyperparathyroidism. All parathyroid gland specimens were cut into pieces and collected in Roswell Park Memorial Institute (RPMI) solution (85 %), dimethyl sulfoxide (DMSO) (10 %), and fetal calf serum (5 %). After step-freezing to −79 °C, specimens were stored in liquid nitrogen (−197 °C). Written informed consent was obtained from all patients.

The parathyroid tissue was thawed in a 37 °C water bath, minced into small fragments in DMEM-f-12 medium (Sigma Chemical, St. Louis, MO, USA), and digested for 2 h at 37 °C in media containing collagenase II (1.2 mg/ml; Sigma Chemical). After centrifugation at 500 g and mechanical dispersion, the pellet was resuspended in complete growth medium (DMEM-F-12) supplemented with 5 % calf serum, 1 % Nutridoma-SP (Boehringer Mannheim, Germany), 100 U penicillin/ml, 100 µg of streptomycin/ml, 1 mM CaCl_2_ and 0.5 mM MgCl_2_, and the suspension was filtered through 60- and 150-mesh screens.

The viability of the detached cells (trypsin/0.06 % EDTA) was tested by the trypan blue method. A mixture of 1 µl of cells (density of 4 × 10^5^/ml) and 5 µl trypan blue was prepared, and then placed in a counting chamber to determine the viability ratio (live cells/live + dead cells).

TheraCyte implantable systems (Irvine, Calif.) were used for cell encapsulation. The parathyroid cells were passed through a 150-mesh screen, collected, and distributed, and then suspended in RPMI solution at a density of 4 × 10^6^ live cells/ml for the TheraCyte group. Using the centrifugation loading method according to the user’s manual (TheraCyte), the TheraCyte-encapsulated live parathyroid cells were implanted into the subcutaneous layer of the necks of the rats. The TheraCyte device consists of an inner membrane of polytetrafluoroethylene (PTFE) that is 30 µm thick and has a 0.4-µm pore size which prevents the entry of cells into the device, while allowing the entry of antibodies and compliment factors, and a laminated 15-µm thick PTFE outer membrane which has a 5-µm pore size that allows improved biocompatibility and induced vascularization. For the final step, 4 × 10^6^ cells in a volume of 1 ml were loaded into the 4.5-µl device (TheraCyte).

### Killing and analysis

Six weeks after surgery, the lumbar spines were excised and the fusion masses were examined. After inspection, manual palpation testing of the L4–L5 segment was performed by three independent observers [17, 18]. Only levels graded as solid by at least two observers were considered to be fused. Posteroanterior radiographs were obtained for all specimens after the rats were killed. The radiographs were viewed and graded as fused or not fused. Representative specimens were fixed in 10 % neutral formalin, decalcified, cut sagittally, then embedded in paraffin, and stained with hematoxylin and eosin for microscopic examination [19, 20]. Blood was drawn at the time of implantation of the TheraCyte implantable system and during killing to measure calcium, phosphorus, and human iPTH levels.

### Statistical analysis

Data were expressed as mean ± standard deviation. Statistical analysis was performed using repeated measures of analysis of variance for iPTH, calcium, and phosphorus levels. Fisher’s exact test (two-tailed) was used to determine if the proportion of rats judged as fused was significantly greater in the TheraCyte group relative to the control group. Comparison of trends in weight gain for the two groups was performed using the one-tailed Student’s *t* test. Significance was defined as *P* < 0.05.

## Results

Two rats expired due to anesthesia toxicity and two rats were excluded from the study due to deep wound infection. The final analysis included 18 rats in the control group and 18 rats in the TheraCyte group. The remaining rats tolerated the surgical procedure well and were ambulatory on the following postoperative days. A trend in weight gain was observed in the rats over time. The increase in body weight for the control and TheraCyte experimental groups was 36.2 ± 18.1 and 34.5 ± 17.1 g, respectively. However, at final examination, there was no significant difference in body weight between the two groups.

**Fig. 1:**
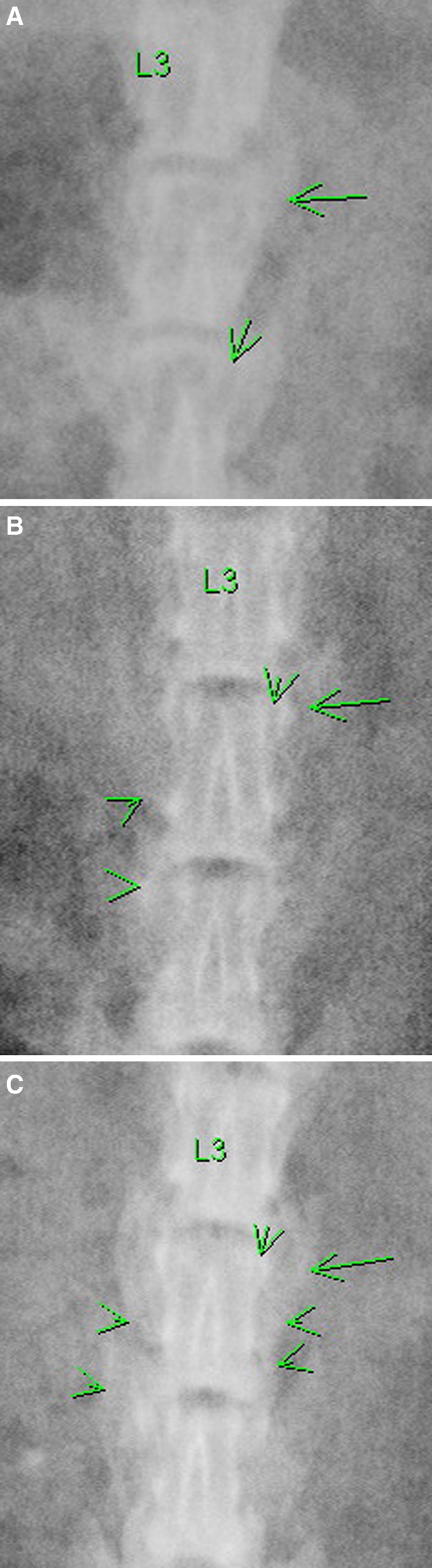
The processes transversus (*long arrow*), facet joint (*short arrow*), fused bony graft (*arrow head*) of lumbar area are *highlighted with arrows*. Typical radiographs of **a** non-fusion, **b** partial fusion, and **c** good fusion are presented

The explanted spines were assessed by manual palpation (Table 1). Only levels graded as solid by at least two observers were considered to be fused. Fusion was found in 6 of 18 rats (33 %) in the control group and 13 of 18 rats (72 %) in the TheraCyte experimental group; the difference was statistically significant (*P* = 0.044). Assessment of the evidence for bone fusion was difficult due to the radiopacity of the rat skeletal system (Fig. 1). The radiographic fusion rate for the control group was 11 % (2/18) and for the TheraCyte group 50 % (9/18).

**Table 1:** Fusion rates (manual palpation data for the control and TheraCyte groups)

	Control (%)	TheraCyte (%)
Observer 1	7/18 (39)	14/18 (78)
Observer 2	7/18 (39)	13/18 (72)
Observer 3	6/18 (33)	11/18 (61)
All*	6/18 (33)	13/18 (72)

* Fisher’s exact test (two-tailed) was used to compare the two groups (*P* = 0.044)

**Fig. 2:**
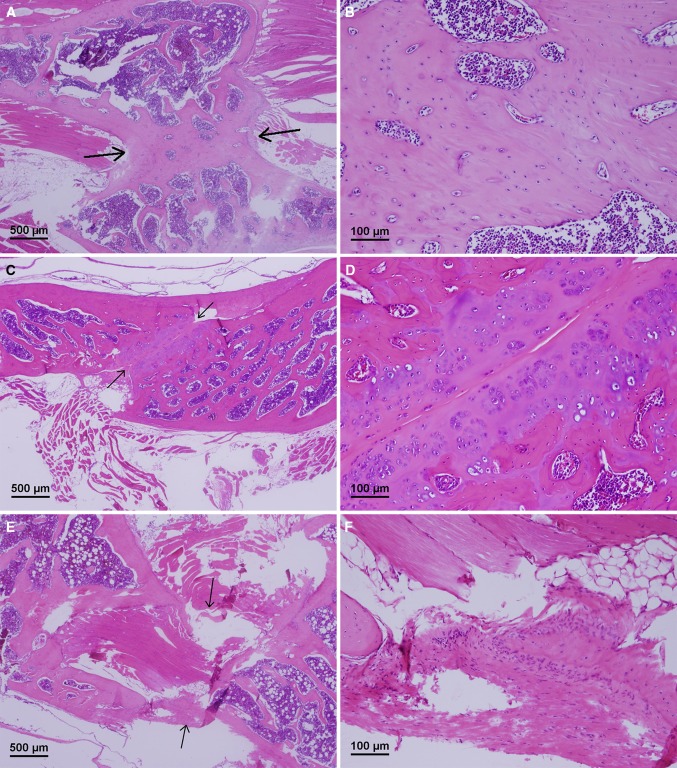
The areas between grafted bony chip are *highlighted with arrows*. Bridging trabeculae (**a**) with new bone formation (**b**) or fibrous adhesion between bone graft chips (**c** and **d**) in the TheraCyte group. Fibroadipose tissue and skeletal muscle separated bone graft chips in the control group (**e** and **f**). Hematoxylin and eosin

Histology analysis revealed bridging trabeculae or fibrous adhesion bone graft chips in the fused specimens (Fig. 2a–d). In the non-fused specimens, the bony islands were widely separated by fibroadipose tissue and skeletal muscle without bridging trabeculae or fibrous adhesion between the graft chips (Fig. 2e, f). Overall, the results of histology analysis were consistent with those of manual palpation. Serum iPTH (pg/ml) levels were significantly higher in the TheraCyte group than in the control group (*P* < 0.05). However, there was no significant difference between the two groups in the level of serum calcium (*P* = 0.118) or phosphorus (*P* = 0.143) (Table 2).

**Table 2:** Serum levels of calcium (mg/dl), phosphorus (mg/dl), and iPTH (pg/ml) at time of implantation and 6 weeks after TheraCyte implantation

	Serum level of calcium*		Serum levels of phosphorus**		Serum levels of iPTH***	
	Time of implantation	6 weeks	Time of implantation	6 weeks	Time of implantation	6 weeks
Control	10.3 ± 0.56	9.6 ± 1.22	5.3 ± 0.35	4.5 ± 0.77	5.45 ± 0.99	4.77 ± 2.1
TheraCyte	10.2 ± 0.55	10.3 ± 0.54	5.2 ± 0.845	5.5 ± 0.43	5.10 ± 1.98	9.3 ± 1.43

Values were obtained using repeated measures of analysis of variance. All data are presented as mean ± standard deviation

* *P* = 0.118

** *P* = 0.143

*** *P* < 0.05

## Discussion

Parathyroid hormone acts via PTH-1 receptors on osteoblasts and bone marrow stromal cells to induce osteoblastic bone formation [19, 21, 22]. Its anabolic effects on the skeleton have been well documented [23–25]. In animal studies, PTH has been shown to facilitate facture healing [7, 23] and increase the lumbar fusion rate [10, 11]. Although effective in the treatment of osteoporosis, daily injection of PTH for a period of 1–2 years is required [8, 24, 26]. However, because daily injection of PTH is inconvenient and troublesome, we used the implantation of parathyroid tissue. In our previous study [14], following implantation of TheraCyte-encapsulated 4 × 10^6^ live parathyroid cells in rats, we found that the parathyroid cells can function very well and increase BMD in the lumbar spine. It was thus hypothesized that the implantation of TheraCyte-encapsulated parathyroid cells might enhance the bone formation in the settings of fracture, non-unions, and fusions. This animal study revealed more fusion in rats that received implantation of TheraCyte-encapsulated 4 × 10^6^ live parathyroid cells than in those rats that did not (72 vs. 33 %). Serum levels of calcium and phosphorus were not significantly different between the two groups. It is presumed that the kidneys of the rats were functioning normally and could keep serum calcium and phosphorus levels within the normal range. Higher serum level of iPTH in the TheraCyte group indicated good viability of the implanted cells while the level was not high enough to cause abnormal calcium concentration.

In the rats in our study, body weight at 6 weeks after implantation was higher than that at baseline. However, the differences between the two groups were not significant and therefore the effect of body weight could be ignored.

Multiple means of fusion assessment have been used in animal studies and fusion rates have differed among the methods of analysis. Manual palpation has been proven to be consistent and predictive of precise multidirectional biomechanical testing in previous studies [18]. While histology provides solid information about bone formation and quality, it is possible that a gross error may miss the bridging bone. Thus, we used manual palpation as the standard to determine whether or not fusion had occurred in this animal model [17, 18]. Radiographic examination has been found to be notoriously poor for determining the presence or absence of fusion in rats due to their small size [17, 18, 27, 28]. In this animal study, we also had difficulty correlating manual palpation results with radiographic findings. In correlation with histology, it appeared that the fibrous adhesion between cartilage was felt as solid fusion on manual palpation while appearing as a gap between implanted bony chips on radiography.

Posterolateral intertransverse process fusion is the common type of surgical fusion performed in the lumbar spine. The reported rate of non-union has ranged from 5 to 35 % [2, 29]. This fusion rate can be improved by systemic medications [10, 11] or bone graft extenders, enhancer, and substitutes [29]. In this rat posterolateral spinal fusion model using the autologous bone graft the TheraCyte-encapsulated live parathyroid cells significantly enhanced posterolateral fusion success. Although the results are encouraging, care must be taken when extrapolating these results to human lumbar fusion. As with any animal study, not all results seen in lower species are reproducible in higher species. Further studies are needed to determine the clinical significance. In conclusion, this pilot animal study revealed more fusions in rats that received TheraCyte-encapsulated 4 × 10^6^ live parathyroid cells without causing high serum calcium or low phosphorus concentrations.
